# Differences in age-related distribution of CSF alpha-synuclein seeding and Alzheimer profiles between PD with and without *GBA1* variants

**DOI:** 10.1038/s41531-025-00978-1

**Published:** 2025-05-05

**Authors:** Stefanie Lerche, Isabel Wurster, Benjamin Roeben, Milan Zimmermann, Marcello Rossi, Angela Mammana, Simone Baiardi, Ann‑Kathrin Hauser, Christian Deuschle, Claudia Schulte, Piero Parchi, Kathrin Brockmann

**Affiliations:** 1 Department of Neurodegeneration, Center of Neurology, Hertie Institute for Clinical Brain Research, German Center for Neurodegenerative Diseases, University of Tuebingen, Hoppe Seyler‑Strasse 3, 72076 Tuebingen, Germany; 2 German Center for Neurodegenerative Diseases, University of Tuebingen, Tuebingen, Germany; 3 Edmond J. Safra Fellow in Movement Disorders, Tübingen, Germany; 4 IRCCS Istituto delle Scienze Neurologiche di Bologna, Via Altura 1/8, 40139 Bologna, Italy; 5 Department of Experimental, Diagnostic and Specialty Medicine (DIMES), University of Bologna, Bologna, Italy

**Keywords:** Biomarkers, Neuroscience, Neurodegeneration, Parkinson's disease

## Abstract

We studied the age- and genetic-related prevalence of CSF alpha-Synuclein seeding and Alzheimer profiles in 188 samples from PD participants without *GBA1* variants (PD_wildtype_) and in 129 samples from PD participants with *GBA1* variants (PD_GBA1_). While Alzheimer profiles increased with age in PD_wildtype_, it was sparse in PD_GBA1_. Alpha-Synuclein profiles remained stable with age in both cohorts. In PD_wildtype_, combined alpha-Synuclein/Alzheimer profiles were associated with earlier onset of cognitive impairment.

The clinicopathological heterogeneity in Parkinson’s disease (PD) highlights the need for pathology-specific in vivo biomarkers. While misfolded and aggregated alpha-synuclein (α-Syn) represents the pathological hallmark of the typical Lewy-body pathology in PD, Alzheimer’s Disease (AD) co-pathology was shown to modify disease progression with regard to the prevalence of and interval until the development of cognitive decline^1,2^. While the classical CSF and blood markers for AD co-pathology such as Amyloid-β and phospho‑Tau species have been widely available for some time, it was only recently that seed amplification assays (SAA), detecting misfolded α-Syn in CSF and other biospecimens have been successfully implemented^3^. Therefore, it is now possible to investigate the underlying pathologies in PD participants in vivo in more detail. The isolated and/or combined prevalence of pathological α-Syn and AD biomarker profiles offer the opportunity to characterize the various CSF pathologies underlying disease progression as a basis for different clinical trajectories to potentially stratify participants for clinical trials. However, whether the prevalence of an isolated or combined CSF pathology change with age and/or disease progression and whether CSF pathology profiles differ between PD participants with and without genetic variants is unclear.

Participants with PD who carry variants in the gene *glucocerebrosidase* (PD_GBA1_) are known to prominently manifest with cognitive decline early on^4–8^. Recently, two large studies could show that PD_GBA1_ present with higher α-Syn seeding activity compared to those without these mutations (PD_wildtype_)^9,10^, Therefore, we aimed to study the age-related prevalence of CSF α-Syn and AD pathology based on CSF biomarker profiles of α-Syn seeding, Amyloid-β_1‑42_, and phospho181‑Tau across different age groups in 188 CSF samples from PD_wildtype_ and in 129 CSF samples from PD_GBA1_ cross-sectionally. We further evaluated the effect of an isolated and combined CSF α-Syn and AD profile on the development of cognitive impairment in 190 longitudinally followed participants.

## Cross-sectional findings

**Fig. 1:**
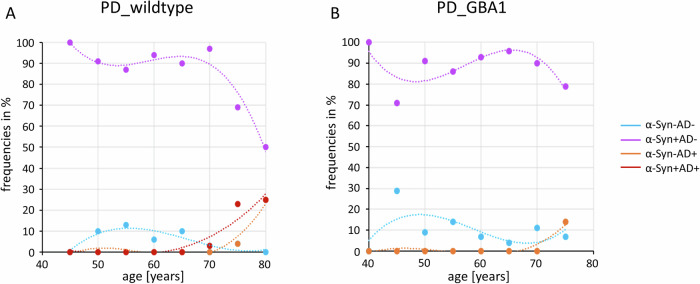
Distribution of CSF α-Syn and AD pathology during age in PD_wildtype_ and PD_GBA1_. Panel A + B show the age-related distribution of isolated or combined CSF α-Syn and AD profiles in PD_wildtype_ (**A**) and PD_GBA1_ (**B**).

The age-related distribution of CSF α-Syn and AD profiles and their combinations is shown in Fig. 1A–B, with additional genetic characteristics of *GBA1* and *APOE4* distribution in Supplemental Table 1.

### PD_wildtype_

Among CSF samples from PD_wildtype_, 175 (93%) were positive for α-Syn. CSF-based AD profiles were identified in 10 (5%) samples. Higher prevalence of AD profiles were associated with increased age (*p* ≤ 0.001) while CSF α-Syn profiles remained stable (*p* = 0.434; Supplemental Fig. 1A). The combination of CSF α-Syn/AD profiles were associated with increased age (*p* ≤ 0.001), Fig. 1A.

Age had an odds ratio of 1.656 (95% confidence interval 1.257-2.180) for AD profiles (*p* ≤ 0.001). No significant effect was seen for age on α-Syn profiles.

### PD_GBA1_

Among CSF baseline samples from PD_GBA1,_ 117 (91%) showed a positive α-Syn profile. All (100%) PD_GBA1_ classified as severe showed CSF α-Syn profiles. Of the 12 samples with negative α-Syn profiles, 7 were risk and 5 mild PD_GBA1_ mutation carriers. None of the CSF samples from all 129 PD_GBA1_ showed a combined α-Syn and AD profil. Isolated AD profiles were identified in 2 (2%) samples of PD_GBA1_ with the mild N370S variant.

A detailed overview of CSF profiles stratified by *GBA1* variant severity (risk, mild, severe) is given in Supplemental Table 1. The distribution of single and combined pathological CSF profiles (α-Syn, AD) did not differ between age groups (*p* > 0.05 respectively), Fig. 1B and Supplemental Fig. 1B.

## Longitudinal findings

Of the 190 longitudinally analyzed PD participants, 57 had cognitive impairment at baseline (PD_GBA1_: n = 31, 54%) and have therefore been excluded from the longitudinal analysis.

Of the 133 PD participants without cognitive impairment at baseline (PD_GBA1_: n = 57), 46 (35%) developed cognitive impairment during study (PD_GBA1_: n = 21, 37%). Participants developing cognitive impairment during the study reached this milestone at a mean study duration of 2.1 years with a mean disease duration of 7.9 years, whereas participants without development of cognitive impairment during the study were followed-up for a mean study duration of 1.7 years until a mean disease duration of 6.8 years (group comparison: study duration p = 0.319; disease duration p = 0.149).

### PD_wildtype_

**Fig. 2:**
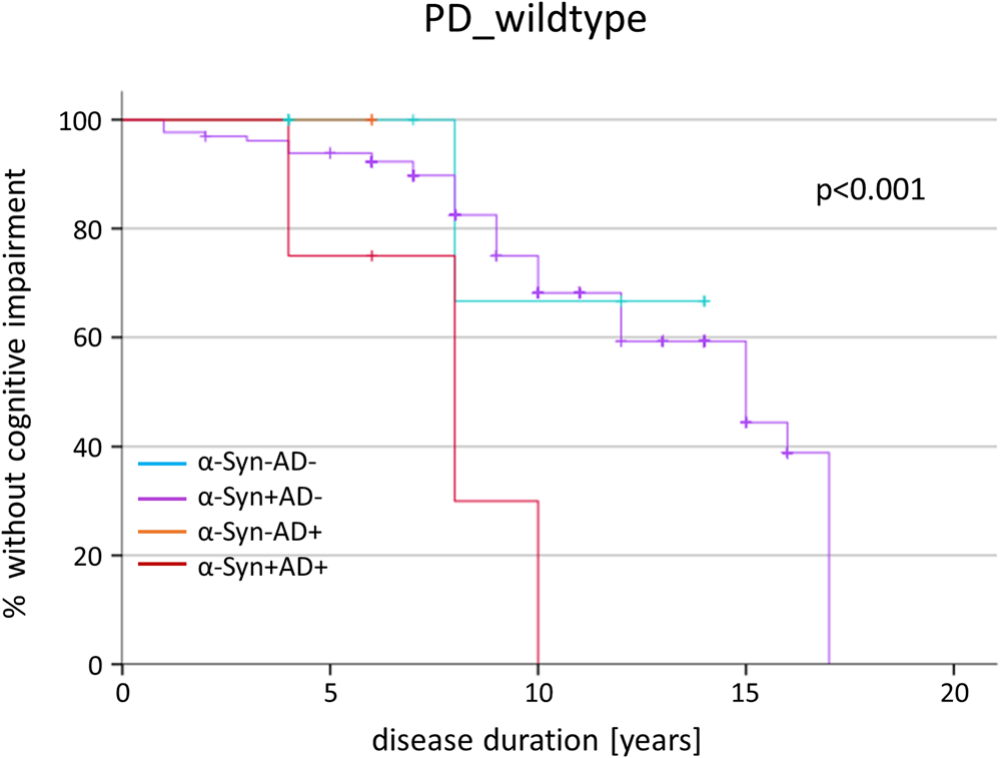
Kaplan-Meier curves and regression analyses depicting the longitudinal development until cognitive impairment stratified by CSF pathology in PD_wildtype_. Kaplan-Meier analyses in PD_wildtype_ revealed a significant effect of pathology on the duration until development of cognitive impairment. Participants with a combined CSF α-Syn/AD pathology reached this milestone earlier in the disease course than those with isolated α-Syn pathology (*p* ≤ 0.001) or without any pathology (*p* = 0.011). Number of PD_wildtype_: α-Syn-/AD- n = 10, α-Syn + /AD- n = 131, α-Syn-/AD+ n = 2, α-Syn + /AD+ n = 8. *Note: Since none of the PD*_*GBA1*_ *showed a combined CSF α-Syn/AD pathology, no meaningful comparison between pathology groups using Kaplan-Meier survival curves was possible*.

Kaplan-Meier survival curves with Mantel-Cox regression revealed that participants with a combined CSF α-Syn/AD profile reached the milestone cognitive impairment faster than participants with isolated α-Syn profile (*p* ≤ 0.001), Fig. 2.

### PD_GBA1_

Since none of the PD_GBA1_ patients showed a combined CSF α-Syn/AD profile, no meaningful group-comparison between CSF profiles using Kaplan-Meier survival curves with Mantel-Cox regression analysis was possible.

## Discussion

The present results indicate a difference in the distribution of age-related CSF biomarkers of α-Syn seeding and AD profiles between PD_wildtype_ and PD_GBA1_. While CSF AD profiles were observed in PD_wildtype_ at ages 75 and older, PD_GBA1_ did not show relevant CSF AD profiles across the age spectrum. Notably, CSF α-Syn profiles remained stable during age in both PD cohorts.

Our present in vivo CSF findings seem in line with a recent post-mortem neuropathology study in 943 cases with Lewy-body disease of which 165 carried a *GBA1* variant. The study showed that *GBA1* variants were most common in cases with a combination of diffuse Lewy-body pathology and a low Braak neurofibrillary tangle stage (0-II) and a low Thal amyloid phase(0-1) highlighting greater Lewy-body and lower AD pathology in PD_GBA1_ than in PD_wildtype_. Interestingly, *GBA1* subgroup analysis revealed that PD_GBA1_ with the severe L483P variant presented with the lowest odds ratio for neurofibrillary tangle and amyloid pathology^11^. These histopathological findings mirror the findings from our present CSF study. Whereas some PD_GBA1_ with risk or mild variants showed CSF profiles of either Amyloid-β or Tau (stable over age), PD_GBA1_ participants with severe variants were 100% CSF α-Syn positive without AD profiles. These findings warrant further investigations with stratification by single *GBA1* variants.

In the group of PD_wildtype_, we could further show that the presence of a combined CSF α-Syn/AD profile is associated with faster cognitive decline compared to isolated α-Syn pathology. This phenomenon is in line with findings from neuropathological studies in Lewy-body cohorts^12,13^. Interestingly, two recent in vivo studies in a memory-clinic cohort with cognitively impaired individuals and in a cohort of clinically unimpaired individuals also reported combined CSF α-Syn/AD profiles to promote the fastest rates of cognitive decline in global cognition as well as in specific domains (attention, executive, memory, visuospatial)^14,15^. Since none of the PD_GBA1_ participants showed a combined CSF α-Syn/AD profile, we were not able to run a longitudinal analysis for the development of cognitive decline stratified by different CSF profiles.

We acknowledge that missing measurements of CSF Aβ_1-40_ poses a limitation. Given the well-established interaction between Aβ_1-42_ and Aβ_1-40_ in β-amyloid fibril formation future studies should include both species. While the difference of follow-up time and disease duration was small, it might pose a limitation for the longitudinal analysis in case some cognitively unimpaired patients may develop cognitive impairment after the last clinical visit.

Strengths of our study include the large monocentric standardized collection of CSF samples, which minimizes variance in sample collection and processing and the complete screening of the *GBA1* gene.

We conclude that stratification by age, CSF pathology profiles and genetic status will be important for future clinical trials addressing cognition.

## Materials and methods

### Participants and clinical investigation

Between 2005 and 2020, 317 CSF samples from PD patients were collected at the University Hospital of Tübingen. All participants were examined by a movement disorders specialist. Diagnosis of PD was defined according to UK Brain Bank Society Criteria^16^. Cognitive function was tested with Montreal Cognitive Assessment (MoCA)^17^ and/or Mini Mental Status Examination (MMSE)^18^. Since the MoCA was available only from 2009 onwards, previously obtained MMSE scores were converted into MoCA equivalents^19^. Cognitive impairment was defined according to criteria reported by Hoops et al. (MoCA ≤25; point of maximum combined sensitivity and specificity)^17^. Distribution of male and female sex was comparable between PD_wildtype_ (70% male) and PD_GBA1_ (73% male). PD_GBA1_ were younger at age at onset (54 years vs. 60 years), younger at lumbar puncture (63 years vs. 66 years) and had a longer disease duration (8 years vs. 6 years) compared to PD_wildtype_. Detailed demographic and clinical information for every CSF sample are listed in Supplemental Table 2.

### Genetic analysis

Genetic screening for pathogenic variants in the gene *GBA1* were done as previously described^20^. GBA1-subgroup classification of variant severity was based on established genotype risks reported for PD. Some variants that have been reported as nonrelevant for Gaucher disease have been proven to increase the risk for PD and therefore have been included in our analysis, for example, p.E326K and p.T369M. We added this information to the methods section^21^. Screening for the presence of *APOε4* allele was done as described in Ref. ^22^.

### CSF collection

Spinal tap was performed between 9.00 am and 1.00 pm. Samples were centrifuged within 60 min and frozen at −80 °C within 90 min after collection. Samples with abnormal routine CSF diagnostics (erythrocytes > 1/μl, white blood cell count > 5 cells/μl, immunoglobulin subtype G index > 0.7) were excluded.

### Purification of recombinant wild-type α-Syn

Purification of recombinant wild-type α-Syn was performed by the following method. Transformed *Escherichia coli* BL21 (DE3) bacteria (New England Biolabs, C2527H) from a glycerol stock were streaked on a selective plate containing kanamycin (Kan + , 50 µg ml^–1^, Sigma, 60615) and incubated at 37 °C overnight. A single colony was selected and inoculated into 5 ml of Luria broth (LB, Sigma, L3397) with kanamycin and allowed to grow for 4–5 h at 37 °C with continuous agitation at 250 rpm. This starter culture was then added to 1 l of LB containing kanamycin and the overnight express autoinduction system (Merk-Millipore, 71300-4) in a fully baffled flask. Cells were grown in a shaking incubator at 37 °C, 200 rpm overnight. The following day the culture was split into four 250 ml flasks and centrifuged at 3200 *g* for 10 min at 4 °C. The pellet was gently resuspended in 25 ml of osmotic shock buffer containing 40% sucrose (Sigma, 84097), 2 mM EDTA (Sigma, *03677*) and 30 mM Tris (Bio-Rad, 1610719) at pH 7.2 using a serological pipette, and incubated for 10 min at room temperature under mild agitation on a rotator mixer. The solution was then centrifuged at 9,000 *g*, 20 min at 20 s and 20 µl of saturated MgCl_2_ (Sigma, M8266) added. After 3 min incubation under mild rocking on ice the suspension was centrifuged at 9,000 *g* for 30 min at 4 °C and the supernatant collected into a 100 ml glass beaker. pH was reduced to 3.5 by the addition of 400–600 µl HCl 1 M (PanReac AppliChem, 13020.1211) and incubated under stirring for 10 min at room temperature. After a second centrifugation at 9,000 *g* for 30 min at 4 °C, the supernatant was collected into a clean 100 ml glass beaker. pH was adjusted to 7.5 by the addition of 400–600 µl of NaOH 1 M (Sigma, 306576). The protein extract was filtered through a 0.22 µm filter (Merk-Millipore, SLGS033SS), loaded into a Ni–NTA column (Cytiva, no. 175248021) on an NGC chromatography system (Bio-Rad) and washed with 20 mM Tris pH 7.5 at room temperature. The column was further washed with 50 mM imidazole (Sigma, i2399) in Tris 20 mM pH 7.5, generating a peak that was not collected. A linear gradient up to 500 mM imidazole in 20 mM Tris pH 7.5 was performed, and the peak collected between 30 and 75% of imidazole buffer (150 and 375 mM, respectively). This peak was loaded onto a Q-HP anion exchange column (Cytiva, 17115401) and washed in Tris 20 mM pH 7.5, followed by another washing in 100 mM NaCl in Tris 20 mM pH 7.5. Again, a linear gradient up to 500 mM of NaCl in Tris 20 mM pH 7.5 was carried out to collect the peak between 300 and 350 mM NaCl. The fractions were pooled, filtered through a 0.22 µm filter and dialyzed against Milli-Q water overnight at 4 °C using a 3.5 kDa MWCO dialysis membrane (Fisher-Scientific, 10005743). The following day, the protein was moved into fresh Milli-Q water and dialyzed for a further 4 h. Protein concentration was measured by spectrophotometry using a theoretical extinction coefficient at 280 nm of 0.36 (mg/ml)^-1^ cm^-1^ and the final solution containing the purified α-Syn was aliquoted in order to have 0.5 mg of α-Syn per tube. Finally, the protein was lyophilized for 6 h and stored at −80 °C until use. The recombinant α-Syn was eventually resuspended into 0.5 ml of phosphate buffer (PB, 40 mM, pH 8.0, Carlo Erba, 369257 and 369143) immediately before the execution of the α-Syn SAA. The a-Syn substrate carries a hexahistidine tag, it is the same substrate as used in the original paper by Groveman et al. ^23^.

### CSF α-Syn seed amplification assay (SAA)

CSF α-Syn seed amplification experiments were performed at the Laboratory of Neuropathology of the Institute of Neurological Science of Bologna (LabNP at ISNB). The assay showed a high specificity (98.7%) for Lewy body pathology in a series of 121 CSF samples from individuals referred to the LabNP at ISNB for dementia of various aetiologies in which the presence of Lewy-related abnormal α-Syn deposits was excluded by neuropathological examination^24,25^.

Briefly, six 0.8 mm silica beads (OPS Diagnostics, BMBG 800-200-02) per well were preloaded into black, clear-bottom, 96-well plates (Thermo Scientific, 265301). CSF samples were thawed and vortexed 10 s before use. Fifteen microliters of CSF were added to 85 μl of a reaction mix composed of 40 mM PB pH 8.0 (Carlo Erba, 369257 and 369143), 170 mM NaCl (Sigma, S9888), 10 µM thioflavin-T (Sigma, T3516), 0.0015% SDS (Bio-Rad, 1610301) and 0.1 g l^–1^ filtered recombinant α-Syn (100-kDa Amicon centrifugal filters, Merck Millipore UFC510096). Plates were closed with a plate sealer film (Thermo Scientific, 232702) and incubated into a Fluostar Omega plate reader (BMG Labtech) at 42 °C with intermittent double-orbital shaking at 400 rpm for 1 min, followed by 1 min rest. Fluorescence was measured every 45 min with 450 nm excitation and 480 nm emission filter. In addition to a negative control, we ran the same positive sample throughout all experiments to optimize the comparison between fluorescent responses in different plates. As negative controls we used CSF samples from patients with normal pressure hydrocephalus which gave a negative result in two SAA runs. The positive control was a CSF sample from a patient with normal pressure hydrocephalus, which gave at least 3 of 4 positive replicates in two SAA runs. To overcome batch-to-batch variations and intrinsic plate-to-plate variability, we normalized within each plate the relative fluorescent units (RFU) of the tested samples at each time point according to the fluorescence peak reached by the positive control by expressing the RFU values as percentages of the maximum fluorescence intensity (i.e., highest RFU value) of the positive control, which we considered 100%.

Each CSF sample was run in quadruplicate and deemed positive when at least 2 out 4 replicates crossed the threshold. We calculated the threshold as the average normalized fluorescence value of negative control repeats during the first 10 h of recording plus 30 standard deviations. The cut-off was set at 30 h. When only one of the four replicates crossed the threshold, the analysis was considered “unclear” and repeated up to three times. In those participants who showed a positive CSF α-Syn seeding profile, we measured the individual mean LAG (time required to reach the threshold; mean out of all positive replicates), the individual mean peak of the fluorescence response (Imax, mean out of all positive replicates), and the individual mean area under the curve (AUC; mean out of all positive replicates). Inter-assay coefficients of variability were < 12% for all three kinetic parameters (LAG 7.7%, Imax 6.8%, AUC 11.8%). For the fluorescence curves see Supplemental Fig. 2.

All experiments were done, and results were reported blinded to the clinical diagnosis and genetic status.

### CSF measurement of Amyloid-β_1‑42_ and phospho181‑Tau

CSF levels of Amyloid-β_1-42_ and phospho181-Tau were measured using ELISA kits from INNOTEST, Fujirebio GmbH, Germany. Intra-assay coefficients of variation for Amyloidβ_1-42_ and phospho181-Tau were below 15%. Our validated in-house cut-offs for clinical routine indicating pathological levels are as follows: Amyloid-β_1-42_ = < 600 pg/ml and phospho181-Tau > 60 pg/ml.

### Definition of pathologies

Amyloid-β pathology profiles were determined by CSF Amyloid-β_1-42_ (cut-off < 600 pg/ml), tau pathology profiles by CSF phospho181-Tau (cut-off phospho181-Tau > 60 pg/ml) and α-Syn pathology profiles by CSF α-Syn seeding positivity. Positivity for CSF AD pathology profiles were defined as having both, pathological CSF Amyloid-β_1-42_ and phospho181-Tau biomarker status according to the criteria of the National Institute of Aging-Alzheimer’s Association^26^. Thereby, **the definition of pathologies solely refers to the CSF biomarker status**, not clinical diagnosis.

### Statistical analysis

Statistical analysis was performed using IBM SPSS 26.0 software. Group comparisons of dichotomous data were analyzed using the likelihood-ratio chi-square test. Intergroup comparisons of demographic and clinical characteristics were calculated using ANOVA. For analysis of pathologies during age, participants were grouped by age at lumbar puncture (five-year intervals combined). Odds ratio calculation was done by logistic regression with age as independent variable and CSF pathology profile as dependent variable.

The effect of different CSF pathology groups on longitudinal development of cognitive impairment was analyzed by Kaplan-Meier survival curves. P- values ≤ 0.05 were considered statistically significant.

### Ethics approval

The study was approved by the Ethics Committee of the University of Tuebingen (26/2007BO1, 404/2010BO1, 199/2011BO1, 702/2013BO1). The study was conducted in accordance with the 1964 Declaration of Helsinki and its later amendments. Written informed consent was obtained from all participants included in the study.

## Supplementary information

Supplemental_tables_figures_NPJ

## Data Availability

Anonymized data are available upon request to: kathrin.brockmann@uni-tuebingen.de.
