# Supplementary material for: Differences in age-related distribution of CSF alpha-synuclein seeding and Alzheimer profiles between PD with and without GBA1 variants
Source: NPJ Parkinsons Dis. 2025 May 5;11:107. doi: 10.1038/s41531-025-00978-1 (PMC12052968; doi:10.1038/s41531-025-00978-1)
Supplement: Supplementary file 1 — Supplemental_tables_figures_NPJ [file 41531_2025_978_MOESM1_ESM.pdf]

Supplemental Table 1: GBA1 and APOε4 variant distribution among age and pathology groups

		**α-Synuclein- AD-** PD$_{wildtype}$ n=12 PD$_{GBA1}$ n=12		**α-Synuclein+ AD-** PD$_{wildtype}$ n=166 PD$_{GBA1}$ n=115		**α-Synuclein- AD+** PD$_{wildtype}$ n=2 PD$_{GBA1}$ n=2		**α-Synuclein+ AD+** PD$_{wildtype}$ n=8 PD$_{GBA1}$ n=0	
Age in years	*GBA1* Genotype	No APOε4	At least 1 APOε4	No APOε4	At least 1 APOε4	No APOε4	At least 1 APOε4	No APOε4	At least 1 APOε4
40	Wildtype: n=0	0 %	0 %	0 %	0 %	0 %	0 %	0 %	0 %
	Risk: n=1	0 %	0 %	100% n=1	0 %	0 %	0 %	0 %	0 %
	Mild: n=0	0 %	0 %	0 %	0 %	0 %	0 %	0 %	0 %
	Severe: n=6	0 %	0 %	100% n=6	0 %	0 %	0 %	0 %	0 %
45	Wildtype: n=3	0 %	0 %	66% n=2	33% n=1	0 %	0 %	0 %	0 %
	Risk: n=2	100% n=2	0 %	0 %	0 %	0 %	0 %	0 %	0 %
	Mild: n=1	0 %	0 %	100% n=1	0 %	0 %	0 %	0 %	0 %
	Severe: n=4	0 %	0 %	75% n=3	25% n=1	0 %	0 %	0 %	0 %
50	Wildtype: n=21	10% n=2	0 %	76% n=16	14% n=3	0 %	0 %	0 %	0 %
	Risk: n=4	0 %	0 %	50% n=2	50% n=2	0 %	0 %	0 %	0 %
	Mild: n=3	0 %	33% n=1	0 %	66% n=2	0 %	0 %	0 %	0 %
	Severe: n=4	0 %	0 %	100% n=4	0 %	0 %	0 %	0 %	0 %
55	Wildtype: n=23	13% n=3	0 %	74% n=17	13% n=3	0 %	0 %	0 %	0 %
	Risk: n=8	13% n=1	0 %	63% n=5	25% n=2	0 %	0 %	0 %	0 %
	Mild: n=2	0 %	100% n=2	0 %	0 %	0 %	0 %	0 %	0 %
	Severe: n=10	0 %	0 %	70% n=7	30% n=3	0 %	0 %	0 %	0 %
60	Wildtype: n=34	6% n=2	0 %	59% n=20	35% n=12	0 %	0 %	0 %	0 %
	Risk: n=14	0 %	0 %	79% n=11	21% n=3	0 %	0 %	0 %	0 %
	Mild: n=7	0 %	28% n=2	14% n=1	57% n=4	0 %	0 %	0 %	0 %
	Severe: n=6	0 %	0 %	66% n=4	33% n=2	0 %	0 %	0 %	0 %
65	Wildtype: n=39	5% n=2	5% n=2	59% n=23	31% n=12	0 %	0 %	0 %	0 %
	Risk: n=15	6% n=1	0 %	87% n=13	6% n=1	0 %	0 %	0 %	0 %
	Mild: n=4	0 %	0 %	25% n=1	75% n=3	0 %	0 %	0 %	0 %
	Severe: n=5	0 %	0 %	80% n=4	20% n=1	0 %	0 %	0 %	0 %
70	Wildtype: n=39	0 %	0 %	82% n=32	15% n=6	0 %	0 %	0 %	3% n=1
	Risk: n=13	15% n=2	0 %	69% n=9	15% n=2	0 %	0 %	0 %	0 %

	Mild: n=1<br>Severe: n=5	0 %<br>0 %	0 %<br>0 %	100% n=1<br>80% n=4	0 %<br>20% n=1	0 %<br>0 %	0 %<br>0 %	0 %<br>0 %	0 %<br>0 %
75	Wildtype: n=26<br>Risk: n=7<br>Mild: n=5<br>Severe: n=2	0 %<br>14% n=1<br>0 %<br>0 %	4% n=1<br>0 %<br>0 %<br>0 %	54% n=14<br>86% n=6<br>40% n=2<br>100% n=2	15% n=4<br>0 %<br>20% n=1<br>0 %	0 %<br>0 %<br>40% n=2<br>0 %	4% n=1<br>0 %<br>0 %<br>0 %	8% n=2<br>0 %<br>0 %<br>0 %	15 % n=4<br>0 %<br>0 %<br>0 %
80	Wildtype: n=3<br>Risk: n=0<br>Mild: n=0<br>Severe: n=0	0 %<br>0%<br>0 %<br>0 %	0 %<br>0%<br>0 %<br>0 %	50% n=2<br>0 %<br>0 %<br>0 %	0 %<br>0 %<br>0 %<br>0 %	0 %<br>0 %<br>0 %<br>0 %	25% n=1<br>0 %<br>0 %<br>0 %	0 %<br>0 %<br>0 %<br>0 %	25% n=1<br>0 %<br>0 %<br>0 %

Two samples with *GBA1* mild variant (age group 55 and age group 60) have no data on APOε4 genotype. Both samples show CSF α-Syn+AD- pathology

Supplemental Table 2: Overview of each sample with information on demographics, genotype, CSF SAA, SAA kinetics and CSF AD profiles.

Sample	Sex	Agegroup [years]	Disease duration [years]	Hoehn &Yahr	UPDRS-III	SAA positivity out of 4 runs	AUC	IMAX	LAG	CSF based pathology_1	CSF based pathology_2	GBA variants	ApoE variants
1	m	40	9,5	2	28	3	670	70,08	22,75	AD-SAA+	A-T-SAA+	p.E365K	E3/E3
2	m	40	5,1	1	19	3	519,4	57,92	24,25	AD-SAA+	A-T-SAA+	p.E365K, p.L483P	E2/E3
3	m	40	7,1	2	18	3	649,8	57,06	20,5	AD-SAA+	A+T-SAA+	p.E365K, p.L483P	E2/E3
4	m	40	15,5	2	39	4	1069	71,84	15,94	AD-SAA+	A-T-SAA+	p.L483P	E3/E3
5	m	40	16,7	3	58	4	1336	101,1	14,81	AD-SAA+	A-T-SAA+	p.L483P	E3/E3
6	m	40	5,4	2	28	4	706	65,89	20,25	AD-SAA+	A-T+SAA+	p.L483P	E3/E3
7	m	40	8	2	22	4	685,2	55,99	19,31	AD-SAA+	A-T+SAA+	p.L483P	E3/E3
8	m	45	14,6	2	18	0				AD-SAA-	A-T-SAA-	p.E365K	E3/E3
9	m	45	15,5	2	10	0				AD-SAA-	A-T-SAA-	p.E365K	E3/E3
10	m	45	8,1	2	19	4	667,3	60,87	20,44	AD-SAA+	A-T-SAA+	p.E365K, p.L483P	E2/E3
11	m	45	9,7	3	58	4	558,3	53,45	22,88	AD-SAA+	A-T+SAA+	p.L483P	E3/E3
12	m	45	1,3	1	10	4	863,2	68,03	17,81	AD-SAA+	A+T-SAA-	p.L483P, p.T408M	E3/E4
13	f	45	2,8	2	16	2	554,9	51,64	24	AD-SAA+	A-T-SAA+	p.S310G	E3/E3
14	m	45	1,9	2	30	3	776,6	73,87	20,5	AD-SAA+	A-T-SAA+	p.W223R	E3/E3
15	f	45	5,4	2	19	3	918,8	90	20,25	AD-SAA+	A-T-SAA+	WT	E3/E4
16	m	45	1,3	2	33	4	796,2	67,1	19,88	AD-SAA+	A-T-SAA+	WT	E2/E3
17	m	45	1,5	2	30	2	436,4	51	24,75	AD-SAA+	A-T-SAA+	WT	E3/E3
18	m	50	15	2	32	3	333,1	46,64	27,25	AD-SAA+	A-T-SAA+	p.D179H, p.E365K	E3/E4
19	m	50	15,9	2	27	4	515,2	60,09	23,44	AD-SAA+	A+T-SAA-	p.D179H, p.E365K	E3/E4
20	m	50	14,3	2	20	4	905,4	49,04	12,75	AD-SAA+	A-T-SAA+	p.E365K	E3/E3
21	m	50	9	2	18	4	624,7	59,54	20,81	AD-SAA+	A-T-SAA+	p.E365K	E3/E3
22	m	50	2,7	2	18	4	1134	93,2	16,5	AD-SAA+	A+T-SAA-	p.E365K	E3/E4
23	m	50	3,5	2	16	4	927,4	71,39	17,63	AD-SAA+	A+T-SAA-	p.L483P	E3/E3
24	m	50	3,8	2	31	4	899,6	66,93	18	AD-SAA+	A-T-SAA+	p.L483P	E3/E3

25	m	50	6,7	2	29	3	597,3	54,82	23,5	AD-SAA+	A-T-SAA+	p.L483P	E3/E3
26	f	50	2,1	1	12	4	684,7	60,56	20,25	AD-SAA+	A-T-SAA+	p.L483P	E3/E3
27	m	50	9,1	2	17	0				AD-SAA-	A-T-SAA-	p.N409S	E3/E4
28	f	50	2,3	2	21	4	1172	79,44	16,31	AD-SAA+	A+T-SAA-	p.T408M	E3/E4
29	m	50	0,3	2	27	2	861,9	82,6	19,5	AD-SAA+	A-T-SAA+	WT	E3/E3
30	f	50	3,4	2	13	1				AD-SAA-	A-T-SAA-	WT	E3/E3
31	f	50	2,3	2	38	3	782,7	66,12	19,5	AD-SAA+	A+T-SAA+	WT	E3/E4
32	m	50	3,3	2	26	3	470,2	48,93	24,75	AD-SAA+	A-T-SAA+	WT	E3/E3
33	m	50	3,5	2	32	3	605,5	61,2	21,94	AD-SAA+	A-T-SAA+	WT	E2/E3
34	f	50	9,4	2	18	3	587,4	60,2	21,5	AD-SAA+	A-T+SAA+	WT	E3/E4
35	m	50	1,8	1	22	4	1027	79,7	17,8	AD-SAA+	A-T-SAA+	WT	E3/E3
36	m	50	3,8	2	25	4	597,6	65,4	22,31	AD-SAA+	A-T-SAA+	WT	E3/E3
37	m	50	2,8	2	21	2	682,2	67,7	21,38	AD-SAA+	A-T-SAA+	WT	E2/E3
38	m	50	5	2	25	2	586,1	78,3	23,25	AD-SAA+	A-T-SAA+	WT	E2/E3
39	m	50	6,3	2	43	1				AD-SAA-	A-T-SAA-	WT	E2/E3
40	f	50	1,8	2	40	4	446,2	59,4	24,75	AD-SAA+	A-T-SAA+	WT	E3/E3
41	f	50	2,9	1	24	4	778,8	80,1	20,25	AD-SAA+	A-T-SAA+	WT	E3/E3
42	m	50	4,5	2	18	2	596	64,7	22,88	AD-SAA+	A+T-SAA+	WT	E3/E3
43	m	50	2,1	2	14	4	661,3	72,5	22,69	AD-SAA+	A+T-SAA-	WT	E3/E4
44	f	50	5,8	2	24	3	905,2	79,5	18,75	AD-SAA+	A-T-SAA+	WT	E2/E3
45	m	50	3,1	2	27	2	658,7	74,3	21,38	AD-SAA+	A-T-SAA+	WT	E3/E3
46	m	50	1,8	2	14	3	871,2	78,7	18,75	AD-SAA+	A+T-SAA+	WT	E3/E3
47	m	50	4,3	2	19	4	940,8	73,2	17,25	AD-SAA+	A-T-SAA+	WT	E3/E3
48	f	50	1,4	1	14	3	429,6	57,2	25,25	AD-SAA+	A-T-SAA+	WT	E3/E3
49	f	50	3,7	2	27	3	480,1	44,1	23,75	AD-SAA+	A-T+SAA+	WT	E3/E3
50	m	55	19,5	2	35	4	787,9	71,1	19,13	AD-SAA+	A-T-SAA+	p.D448H	E3/E4
51	m	55	7,2	2	22	4	604,7	65,54	21,94	AD-SAA+	A+T-SAA-	p.E365K	E3/E3
52	m	55	8,2	2	33	4	868,9	79,03	19,13	AD-SAA+	A-T-SAA+	p.E365K	E3/E3
53	m	55	3,7	2	29	4	889,7	67,3	17,63	AD-SAA+	A+T-SAA-	p.E365K	E4/E4
54	m	55	4,5	2	18	4	791,3	66,43	19,13	AD-SAA+	A+T-SAA-	p.E365K	E3/E3
55	m	55	6,7	2	12	4	716,1	67,72	20,25	AD-SAA+	A-T-SAA+	p.E365K	E3/E3
56	m	55	6,6	2	48	4	1046	66,75	15	AD-SAA+	A-T-SAA+	p.L483P	E3/E3

57	f	55	5,7	2	26	4	784,4	64,01	18,56	AD-SAA+	A-T-SAA+	p.L483P	E3/E3
58	m	55	7,6	2	32	4	1132	95,61	16,88	AD-SAA+	A+T-SAA-	p.L483P	E3/E3
59	m	55	8,3	2	33	4	1060	90,19	17,63	AD-SAA+	A+T-SAA-	p.L483P	E3/E3
60	m	55	11,5	3	37	4	880,1	70,89	18,19	AD-SAA+	A-T-SAA+	p.L483P	E3/E3
61	m	55	7,4	2	42	3	793,2	61,38	18,5	AD-SAA+	A-T-SAA+	p.L483P	E3/E4
62	m	55	8,6	2	53	4	694	67,45	20,63	AD-SAA+	A+T-SAA-	p.L483P	E3/E4
63	m	55	3,3	2	53	0				AD-SAA-	A-T-SAA-	p.N409S	E3/E4
64	f	55	7	2,5	32	4	624,6	56,9	21	AD-SAA+	A-T-SAA+	p.N409S	-
65	m	55	1,8	2	13	0				AD-SAA-	A+T-SAA-	p.N409S	E3/E4
66	m	55	1,3	2	30	0				AD-SAA-	A-T-SAA-	p.T336S	E3/E3
67	m	55	4,6	2,5	33	3	464,8	56,57	24	AD-SAA+	A-T-SAA+	p.T408M	E3/E4
68	f	55	18	2,5	49	4	1018	85,1	17,63	AD-SAA+	A+T-SAA-	p.T408M	E3/E3
69	m	55	6,3	2	19	4	775,1	67,29	18,94	AD-SAA+	A-T-SAA+	p.W223R	E3/E3
70	m	55	8,1	2	30	3	506,8	58,27	23,5	AD-SAA+	A-T-SAA+	p.W223R	E3/E3
71	m	55	3	2	20	2	627,1	61,8	21,38	AD-SAA+	A+T-SAA+	WT	E3/E3
72	m	55	3,4	2	20	3	845,7	86,1	19,8	AD-SAA+	A+T-SAA+	WT	E2/E4
73	m	55	5,8	2	32	3	301,8	43	27,5	AD-SAA+	A-T-SAA+	WT	E2/E4
74	f	55	2,8	2	19	0				AD-SAA-	A-T-SAA-	WT	E3/E3
75	f	55	6,7	1	16	0				AD-SAA-	A-T-SAA-	WT	E3/E3
76	m	55	8	2	24	4	1015	78,1	16,88	AD-SAA+	A-T-SAA+	WT	E2/E3
77	m	55	5,8	2	28	4	985,3	78,7	17,44	AD-SAA+	A-T-SAA+	WT	E3/E3
78	m	55	7,8	2	32	4	659,8	68,2	21,75	AD-SAA+	A-T-SAA+	WT	E3/E3
79	m	55	8,8	2	27	4	967,2	76,1	17,44	AD-SAA+	A+T-SAA-	WT	E3/E3
80	m	55	8,4	2	29	3	427,6	53,6	25,13	AD-SAA+	A-T-SAA+	WT	E2/E3
81	m	55	1,9	2	23	4	638,5	59,4	23,4	AD-SAA+	A-T-SAA+	WT	E3/E4
82	f	55	3,8	2	27	3	658,4	64,3	22,69	AD-SAA+	A-T-SAA+	WT	E3/E3
83	f	55	5,6	2	29	2	802,2	68,5	19,5	AD-SAA+	A-T-SAA+	WT	E3/E3
84	f	55	3,7	2	16	0				AD-SAA-	A-T-SAA-	WT	E3/E3
85	f	55	5,6	2	16	3	882,5	70,7	18	AD-SAA+	A-T-SAA+	WT	E3/E3
86	f	55	5,6	3	44	3	526,9	63,3	23,75	AD-SAA+	A-T-SAA+	WT	E3/E4
87	m	55	7,6	2	31	3	1320	97,2	15,25	AD-SAA+	A+T-SAA+	WT	E3/E3
88	m	55	4,8	2	25	4	317	43,8	27	AD-SAA+	A+T-SAA-	WT	E3/E3

89	m	55	7,7	3	16	3	709,4	75,8	19,5	AD-SAA+	A-T-SAA+	WT	E3/E3
90	f	55	1,7	1	11	3	688,1	57,9	20,75	AD-SAA+	A-T-SAA+	WT	E2/E3
91	m	55	6,6	2	39	4	856,5	73,6	18,94	AD-SAA+	A-T-SAA+	WT	E3/E3
92	m	55	8,9	2	41	4	719,6	77,7	22,31	AD-SAA+	A-T-SAA+	WT	E3/E3
93	f	55	1,2	1	8	3	350,3	48,8	26,25	AD-SAA+	A+T-SAA+	WT	E3/E4
94	m	60	7,8	3	10	4	1211	90,68	15,75	AD-SAA+	A-T-SAA+	c.1265_1319del, p.D448H, p.L483P	E3/E4
95	m	60	9,8	2	43	4	1179	89,27	15,94	AD-SAA+	A-T-SAA+	p.E365K	E2/E3
96	m	60	11,8	3	36	4	912,9	85,67	21,56	AD-SAA+	A+T-SAA-	p.E365K	E2/E3
97	m	60	6,8	2	32	4	1020	78,86	17,06	AD-SAA+	A+T-SAA-	p.E365K	E3/E3
98	m	60	6,7	2	26	4	721,9	68,53	21,56	AD-SAA+	A-T+SAA+	p.E365K	E3/E3
99	m	60	5,8	1	16	4	1005	89,61	17,63	AD-SAA+	A+T-SAA-	p.E365K	E3/E4
100	m	60	10,2	2	25	4	1152	88,97	17,06	AD-SAA+	A-T-SAA+	p.E365K	E3/E3
101	f	60	12,6	2	24	3	480,3	55,79	24,25	AD-SAA+	A+T-SAA+	p.E365K	E3/E3
102	f	60	13,5	2	37	3	1120	88,29	17	AD-SAA+	A-T-SAA+	p.E365K	E3/E3
103	m	60	2,7	2	16	3	1088	89,97	17,25	AD-SAA+	A+T-SAA+	p.E365K	E3/E4
104	f	60	6,2	2	17	4	845,5	63,32	18	AD-SAA+	A-T+SAA+	p.E365K	E2/E3
105	f	60	8,2	2	10	2	666	55,16	22,88	AD-SAA+	A-T+SAA+	p.E365K	E2/E3
106	f	60	10,2	2	18	4	940	70,52	17,44	AD-SAA+	A-T+SAA+	p.E365K	E2/E3
107	m	60	6,3	2	28	4	1102	90,63	17,63	AD-SAA+	A-T-SAA+	p.L483P	E3/E3
108	m	60	12,8	3	45	4	702,6	64,24	20,63	AD-SAA+	A+T-SAA-	p.L483P	E3/E3
109	m	60	12,7	2	57	4	1184	102,7	17,25	AD-SAA+	A+T-SAA-	p.L483P	E3/E4
110	f	60	10,2	2	43	4	772,7	62,75	19,31	AD-SAA+	A-T-SAA+	p.L483P	E3/E3
111	f	60	7,6	2	26	3	784,9	78,6	20,25	AD-SAA+	A-T-SAA+	p.N409S	E3/E4
112	f	60	11,8	3	39	3	446,3	43,64	24,25	AD-SAA+	A-T-SAA+	p.N409S	E3/E4
113	m	60	15	2	21	4	652	63,64	21,38	AD-SAA+	A-T-SAA+	p.N409S	E3/E4
114	m	60	19,2	2	38	4	612,7	59,97	21,94	AD-SAA+	A-T-SAA+	p.N409S	E3/E4
115	f	60	10,3	2	25	4	1166	75,4	14,63	AD-SAA+	A-T-SAA+	p.N409S	-
116	m	60	3,7	2	12	0				AD-SAA-	A-T-SAA-	p.N409S	E3/E4
117	m	60	5,7	2	30	0				AD-SAA-	A-T-SAA-	p.N409S	E3/E4
118	m	60	11,6	3	26	3	969,2	82,79	18,5	AD-SAA+	A-T-SAA+	p.R398*	E3/E3

119	m	60	5,3	2	39	3	906,3	76,14	18	AD-SAA+	A+T-SAA+	p.S310G, p.N409S	E3/E3
120	f	60	6,7	3	35	4	758,8	66,49	20,63	AD-SAA+	A+T-SAA-	p.T408M	E3/E3
121	m	60	6,1	2	30	3	614,3	74,09	22,5	AD-SAA+	A-T-SAA+	p.T408M	E3/E4
122	m	60	2,5	2,5	14	2	1013	78,5	17,25	AD-SAA+	A-T-SAA+	WT	E3/E3
123	m	60	4,8	2	18	3	828,6	79,4	19,69	AD-SAA+	A+T-SAA+	WT	E3/E3
124	m	60	2,8	2	31	4	710,1	63,4	19,88	AD-SAA+	A+T-SAA-	WT	E3/E3
125	m	60	6,7	2	14	2	687	58,8	19,5	AD-SAA+	A-T-SAA+	WT	E3/E3
126	m	60	7,5	2	31	4	764,6	60,8	18,6	AD-SAA+	A+T-SAA-	WT	E3/E3
127	m	60	5	2	33	4	805,2	72	19,69	AD-SAA+	A-T-SAA+	WT	E3/E3
128	f	60	7,7	3	44	4	673,6	71,9	22,5	AD-SAA+	A+T-SAA-	WT	E3/E3
129	m	60	6,7	2	17	4	813,6	74,1	19,13	AD-SAA+	A+T-SAA-	WT	E3/E4
130	m	60	6,8	2	31	3	573,2	54	21,75	AD-SAA+	A-T-SAA+	WT	E3/E3
131	m	60	6,8	2	11	4	1247	102	16,7	AD-SAA+	A-T-SAA+	WT	E4/E4
132	m	60	13	2	27	3	544,8	55,62	23,5	AD-SAA+	A-T-SAA+	WT	E3/E3
133	f	60	6,8	2	17	0				AD-SAA-	A+T-SAA-	WT	E3/E3
134	m	60	4,6	1	9	4	560,9	60,1	22,88	AD-SAA+	A+T-SAA-	WT	E3/E4
135	m	60	13,4	2	16	2	191	34,43	30	AD-SAA+	A-T-SAA+	WT	E3/E4
136	f	60	12,1	2	10	0				AD-SAA-	A-T-SAA-	WT	E3/E3
137	m	60	9	2	38	3	684,8	62,6	22,5	AD-SAA+	A-T-SAA+	WT	E2/E3
138	m	60	3,9	2	35	3	618,4	62,6	21,5	AD-SAA+	A-T-SAA+	WT	E3/E4
139	m	60	5,8	2	41	4	387,2	51,9	25,88	AD-SAA+	A-T-SAA+	WT	E3/E4
140	m	60	7,8	2	28	3	414,3	48,1	25,5	AD-SAA+	A-T-SAA+	WT	E3/E4
141	m	60	2,8	2	27	3	359,3	48,6	26,75	AD-SAA+	A-T-SAA+	WT	E3/E4
142	m	60	4,9	2	36	4	767,2	70,5	20,63	AD-SAA+	A-T-SAA+	WT	E3/E4
143	f	60	7,8	2,5	28	3	811,8	75,1	19,88	AD-SAA+	A-T-SAA+	WT	E3/E3
144	f	60	8,8	3	38	2	971,8	76,2	17,25	AD-SAA+	A-T-SAA+	WT	E3/E3
145	m	60	5,5	2	24	3	741	69,7	20,06	AD-SAA+	A-T-SAA+	WT	E3/E3
146	f	60	7,5	2	19	3	888,9	70,5	19,5	AD-SAA+	A-T-SAA+	WT	E3/E4
147	f	60	9,6	3	36	4	784,7	64	18,56	AD-SAA+	A+T-SAA-	WT	E3/E4
148	m	60	6,8	2	24	4	693,7	60,3	20,63	AD-SAA+	A+T-SAA-	WT	E3/E3
149	m	60	6,6	2	19	4	1069	95,1	17,63	AD-SAA+	A+T-SAA-	WT	E2/E3

150	m	60	6	2	24	3	228,6	42,1	29	AD-SAA+	A-T-SAA+	WT	E3/E3
151	f	60	1,9	2	17	3	545,3	53,7	21,75	AD-SAA+	A-T-SAA+	WT	E3/E3
152	m	60	1,3	2	28	3	479,8	50,1	23,75	AD-SAA+	A-T-SAA+	WT	E3/E3
153	m	60	4,3	2	32	4	578,1	60,8	23,06	AD-SAA+	A-T-SAA+	WT	E3/E3
154	m	60	4,7	2	5	4	679,8	68,1	20,81	AD-SAA+	A+T-SAA-	WT	E3/E4
155	m	60	4,3	2,5	19	2	743,3	63,6	19,88	AD-SAA+	A-T-SAA+	WT	E3/E3
156	f	65	7,3	2	8	4	941,5	76,88	19,5	AD-SAA+	A+T-SAA-	c.115+1G>A	E3/E4
157	m	65	12,1	2	31	3	1502	96,34	13,5	AD-SAA+	A-T-SAA+	p.E365K	E3/E3
158	m	65	4,3	2	31	3	480,5	55,91	24,38	AD-SAA+	A-T-SAA+	p.E365K	E3/E3
159	f	65	4,1	1	7	4	646,9	63,57	21,94	AD-SAA+	A-T-SAA+	p.E365K	E2/E3
160	m	65	10,8	2	22	4	440	52,83	24,19	AD-SAA+	A-T-SAA+	p.E365K	E2/E3
161	m	65	7,8	2,5	32	4	856,2	81,49	19,88	AD-SAA+	A-T+SAA+	p.E365K	E3/E3
162	m	65	9,9	3	46	4	1122	96,19	17,81	AD-SAA+	A-T+SAA+	p.E365K	E3/E3
163	f	65	1,8	1	30	4	1247	94,59	16,13	AD-SAA+	A-T-SAA+	p.E365K	E3/E4
164	m	65	10,3	2	28	4	758,6	61,15	18,75	AD-SAA+	A+T-SAA-	p.E365K	E3/E3
165	m	65	6,1	3	24	4	1258	102,1	16,13	AD-SAA+	A+T-SAA-	p.E365K	E2/E3
166	m	65	0,8	1	14	0				AD-SAA-	A+T-SAA-	p.E365K	E3/E3
167	f	65	7,9	2	30	4	1137	76,24	15,38	AD-SAA+	A+T-SAA-	p.E365K, p.L483P	E3/E3
168	m	65	9,3	3	47	4	1081	66,54	14,25	AD-SAA+	A-T-SAA+	p.L483P	E3/E3
169	m	65	6,3	2	15	4	682,6	68,95	21,38	AD-SAA+	A-T-SAA+	p.L483P	E3/E3
170	m	65	7,2	2	35	4	894,5	74,29	18,38	AD-SAA+	A+T-SAA-	p.L483P	E3/E3
171	f	65	12,8	3	70	4	552	50,45	21,75	AD-SAA+	A-T-SAA+	p.N409S	E3/E4
172	m	65	8,7	3	43	4	803,2	74,62	20,06	AD-SAA+	A+T-SAA-	p.N409S	E3/E4
173	m	65	20,9	2	12	4	482,9	54,22	24,38	AD-SAA+	A+T-SAA-	p.N409S	E3/E4
174	m	65	5,8	2	26	4	908,5	83,13	18,75	AD-SAA+	A-T-SAA+	p.N409S	E3/E3
175	f	65	4,3	2	13	4	1079	86,3	17,25	AD-SAA+	A-T-SAA+	p.R78C	E3/E3
176	m	65	3,3	2	17	4	532,8	56,09	23,25	AD-SAA+	A-T-SAA+	p.T408M	E3/E3
177	f	65	2,8	3	24	3	761	76,62	22,25	AD-SAA+	A+T-SAA+	p.T408M	E3/E3
178	f	65	4,7	3	20	3	646,1	62,7	21	AD-SAA+	A-T-SAA+	p.T408M	E3/E3
179	m	65	8,5	2	37	4	506,9	64,39	23,81	AD-SAA+	A-T-SAA+	p.T408M	E3/E3
180	f	65	5,4	2	41	4	429,2	52,8	25,75	AD-SAA+	A+T-SAA-	WT	E3/E4
181	m	65	5,3	2	20	4	654,5	60,2	20,63	AD-SAA+	A-T-SAA+	WT	E3/E3

182	m	65	9,9	2	38	3	652,4	75,6	21,75	AD-SAA+	A+T-SAA+	WT	E3/E3
183	m	65	8,4	2	26	4	1281	89,5	15,19	AD-SAA+	A+T-SAA-	WT	E3/E3
184	m	65	5,8	2	32	4	564,1	59,2	22,13	AD-SAA+	A-T-SAA+	WT	E3/E3
185	m	65	10,5	2	29	4	1365	102	15,56	AD-SAA+	A+T-SAA-	WT	E4/E4
186	m	65	12,7	2	20	4	652,2	60,6	20,44	AD-SAA+	A+T-SAA-	WT	E4/E4
187	m	65	13,6	2	26	4	598,1	57,4	21,75	AD-SAA+	A+T-SAA-	WT	E4/E4
188	m	65	5,6	2	7	4	845,1	68	19,1	AD-SAA+	A-T-SAA+	WT	E3/E3
189	m	65	3,7	2	23	2	557,9	68,81	23,25	AD-SAA+	A-T-SAA+	WT	E3/E3
190	m	65	5,7	2	20	3	442,9	64,83	24,5	AD-SAA+	A-T-SAA+	WT	E3/E3
191	m	65	9,2	2	23	4	682,6	62	21	AD-SAA+	A-T-SAA+	WT	E3/E3
192	m	65	12,2	2	26	4	754,6	75,4	20,44	AD-SAA+	A-T-SAA+	WT	E3/E3
193	m	65	13,1	2	27	4	949,6	79,8	18,19	AD-SAA+	A-T-SAA+	WT	E3/E3
194	m	65	1,9	2	23	4	580,1	58,5	23,4	AD-SAA+	A-T-SAA+	WT	E3/E3
195	m	65	3,9	1	16	4	808,3	70	19,69	AD-SAA+	A-T-SAA+	WT	E3/E3
196	f	65	12,3	1	3	0				AD-SAA-	A+T-SAA-	WT	E3/E3
197	m	65	3,9	2	21	3	890,9	85	19	AD-SAA+	A+T-SAA+	WT	E3/E3
198	m	65	7	2	24	4	1013	78,6	18,56	AD-SAA+	A+T-SAA-	WT	E3/E4
199	m	65	3,3	2	23	4	526,7	51,2	22,88	AD-SAA+	A-T-SAA+	WT	E3/E3
200	m	65	6,8	2	25	4	418,4	46,3	25,13	AD-SAA+	A-T-SAA+	WT	E3/E4
201	m	65	7,8	2	29	4	503,4	58,3	23,63	AD-SAA+	A+T-SAA-	WT	E3/E4
202	m	65	9,5	2	30	4	458,2	55,9	25,13	AD-SAA+	A-T+SAA+	WT	E3/E3
203	m	65	11,5	2	25	3	807,4	67,3	18,25	AD-SAA+	A-T+SAA+	WT	E3/E3
204	f	65	5,4	2	10	0				AD-SAA-	A-T+SAA-	WT	E2/E3
205	m	65	18,5	5	42	4	1004	77,7	18	AD-SAA+	A+T-SAA-	WT	E3/E4
206	m	65	4,9	2	37	4	704,8	66,8	20,63	AD-SAA+	A-T-SAA+	WT	E2/E3
207	m	65	2,3	1	25	2	511,5	43,1	22,5	AD-SAA+	A-T-SAA+	WT	E3/E3
208	f	65	1,9	1	5	4	960,8	89,3	20,44	AD-SAA+	A+T-SAA-	WT	E3/E3
209	f	65	2,2	1	9	3	462,6	62,1	24,75	AD-SAA+	A+T-SAA+	WT	E3/E3
210	m	65	6,6	2	12	4	860,1	70,3	18,94	AD-SAA+	A-T-SAA+	WT	E3/E3
211	f	65	5,5	1	4	3	953,8	75,5	17,75	AD-SAA+	A+T-SAA+	WT	E3/E4
212	f	65	7,8	2	27	4	519,9	61	23,81	AD-SAA+	A-T-SAA+	WT	E3/E4
213	m	65	3,7	2	29	4	406,2	55,2	25,5	AD-SAA+	A-T-SAA+	WT	E3/E3

214	m	65	3,6	2	30	4	905,5	89,6	21,19	AD-SAA+	A+T-SAA-	WT	E3/E4
215	m	65	6	2	38	3	461	53,8	25	AD-SAA+	A+T-SAA+	WT	E3/E4
216	f	65	2,3	2	34	0				AD-SAA-	A+T-SAA-	WT	E3/E4
217	f	65	4,4	2,5	33	0				AD-SAA-	A+T-SAA-	WT	E3/E4
218	m	65	3,8	2	23	4	527,9	60,1	23,6	AD-SAA+	A-T-SAA+	WT	E3/E3
219	f	70	3,5	2	12	3	691,6	63,65	20,25	AD-SAA+	A-T+SAA+	c.115+1G>A	E2/E3
220	f	70	6,4	2	25	3	666,2	64,22	21,75	AD-SAA+	A-T-SAA+	p.E365K	E3/E3
221	f	70	1	2	17	2	517,2	67,59	24	AD-SAA+	A-T-SAA+	p.E365K	E2/E3
222	m	70	14,6	2	56	4	767,8	75,45	20,44	AD-SAA+	A+T-SAA-	p.E365K	E3/E3
223	m	70	1,7	1	17	4	1026	90,85	18,19	AD-SAA+	A+T-SAA-	p.E365K	E3/E4
224	m	70	7,6	2	31	2	426,4	55,25	25,5	AD-SAA+	A-T-SAA+	p.E365K	E3/E3
225	m	70	5,4	2	52	4	599	64,92	23,06	AD-SAA+	A-T+SAA+	p.E365K	E3/E3
226	m	70	11,5	2,5	23	4	575,3	59,87	21,94	AD-SAA+	A-T+SAA+	p.E365K	E3/E3
227	m	70	2,3	2	24	3	656,1	68,14	21,38	AD-SAA+	A-T-SAA+	p.E365K	E3/E3
228	m	70	4,3	2	42	3	672,4	68,28	22,75	AD-SAA+	A-T-SAA+	p.E365K	E3/E3
229	m	70	3,3	1	8	0				AD-SAA-	A+T-SAA-	p.E365K	E3/E3
230	m	70	4,8	1	6	0				AD-SAA-	A+T-SAA-	p.E365K	E3/E3
231	m	70	10,7	2	53	4	665,3	63,7	20,81	AD-SAA+	A+T-SAA-	p.G241R	E3/E3
232	m	70	11,3	5	66	4	760,4	61,46	18,75	AD-SAA+	A+T-SAA-	p.L483P	E3/E3
233	f	70	11,4	4	42	4	755,9	79,46	20,81	AD-SAA+	A+T-SAA-	p.L483P	E3/E4
234	f	70	8	2	52	4	1008	79,7	18,19	AD-SAA+	A-T-SAA+	p.L483P	E2/E3
235	f	70	21,6	4	39	4	932,5	92,03	19,31	AD-SAA+	A-T-SAA+	p.N409S	E2/E3
236	m	70	6,4	2	44	4	399,9	48,66	25,75	AD-SAA+	A+T-SAA-	p.T408M	E3/E4
237	m	70	0,4	2	22	4	567,8	51,97	21,94	AD-SAA+	A+T-SAA-	p.T408M	E3/E3
238	f	70	14,7	2	26	4	719,5	65,36	20,44	AD-SAA+	A-T-SAA+	WT	E3/E3
239	f	70	18,8	2	33	4	465,5	48,68	24	AD-SAA+	A-T-SAA+	WT	E3/E3
240	f	70	4,3	2	30	4	980,6	77,6	17,25	AD-SAA+	A+T-SAA-	WT	E3/E4
241	f	70	8,4	2,5	16	4	567,8	51,97	21,94	AD-SAA+	A-T+SAA+	WT	E3/E3
242	f	70	10,3	2,5	33	4	893,3	75,3	18,56	AD-SAA+	A+T-SAA-	WT	E3/E4
243	m	70	2,9	2	16	3	1177	96,5	16,5	AD-SAA+	A+T-SAA+	WT	E2/E3
244	m	70	3,9	2	19	4	631,1	66,7	21,56	AD-SAA+	A-T-SAA+	WT	E2/E3
245	m	70	3,5	2	28	4	1254	97,6	16,31	AD-SAA+	A+T-SAA-	WT	E3/E3

246	m	70	4,6	2	22	2	330,8	44,1	26,63	AD-SAA+	A-T-SAA+	WT	E3/E3
247	m	70	5,2	2	38	4	869,1	82,1	19,31	AD-SAA+	A-T-SAA+	WT	E3/E3
248	f	70	6,8	2	33	4	1061	78	16,69	AD-SAA+	A+T-SAA-	WT	E3/E4
249	m	70	4,7	2	10	4	778,1	76,7	20,06	AD-SAA+	A+T-SAA-	WT	E3/E3
250	m	70	9,8	2	33	4	568,9	79,1	23,81	AD-SAA+	A-T-SAA+	WT	E3/E3
251	m	70	11,8	2	32	4	678,9	64,6	20,63	AD-SAA+	A-T-SAA+	WT	E3/E3
252	m	70	5,5	2	22	4	625,5	72,7	23,06	AD-SAA+	A-T+SAA+	WT	E3/E4
253	m	70	7,5	2	32	4	1085	91,2	17,25	AD-SAA+	A-T+SAA+	WT	E3/E4
254	m	70	11,1	2	30	4	764,2	64,4	19,13	AD-SAA+	A-T-SAA+	WT	E3/E3
255	m	70	7,7	2	15	3	496,1	56,37	23,75	AD-SAA+	A-T-SAA+	WT	E3/E3
256	m	70	8,6	2	28	3	369,2	48,99	26,75	AD-SAA+	A-T-SAA+	WT	E3/E3
257	m	70	10,6	2	21	4	581,8	62,33	22,13	AD-SAA+	A-T-SAA+	WT	E3/E3
258	m	70	2,8	2	20	4	1164	84,5	15,8	AD-SAA+	A-T-SAA+	WT	E3/E3
259	m	70	5,2	2	31	4	684,4	64,4	20,44	AD-SAA+	A-T-SAA+	WT	E3/E3
260	m	70	2,8	1	7	3	655,7	66,1	21,5	AD-SAA+	A-T-SAA+	WT	E3/E3
261	m	70	5,4	2	22	4	644,9	57,7	20,81	AD-SAA+	A-T-SAA+	WT	E3/E3
262	f	70	3,6	2	21	3	656,6	63,6	21,75	AD-SAA+	A-T-SAA+	WT	E3/E3
263	f	70	4,6	2	14	4	736,4	64,2	19,5	AD-SAA+	A-T-SAA+	WT	E3/E3
264	m	70	6	2	25	3	681,8	77,5	22	AD-SAA+	A-T-SAA+	WT	E3/E3
265	m	70	7,9	2	16	3	515,4	54,7	23,5	AD-SAA+	A-T-SAA+	WT	E3/E3
266	m	70	6,5	2,5	33	4	441,5	50,7	24,38	AD-SAA+	A+T-SAA-	WT	E3/E3
267	m	70	5,8	1,5	22	4	900,1	67,3	17,3	AD-SAA+	A-T-SAA+	WT	E3/E3
268	m	70	2,1	2	23	3	687,6	64,7	22,3	AD-SAA+	A-T+SAA+	WT	E3/E3
269	m	70	2,3	2	33	4	1480	101	13,88	AD-SAA+	A-T-SAA+	WT	E3/E3
270	m	70	4,9	2	31	4	885,2	81,1	19,5	AD-SAA+	A-T-SAA+	WT	E2/E3
271	m	70	7,6	2	8	4	722,2	62,2	19,5	AD-SAA+	A-T-SAA+	WT	E2/E3
272	m	70	6,9	2	19	3	1217	91,8	15,75	AD-SAA+	A-T-SAA+	WT	E3/E3
273	m	70	6,4	2	26	4	601,9	60,1	21,75	AD-SAA+	A-T+SAA+	WT	E3/E4
274	f	70	4,7	2	11	3	611,7	67,7	22,75	AD-SAA+	A-T+SAA+	WT	E3/E3
275	m	70	6	2	16	4	1133	88	17,06	AD+SAA+	A+T+SAA+	WT	E4/E4
276	m	70	5,8	2	27	4	1132	81,4	15,94	AD-SAA+	A+T-SAA-	WT	E3/E3

277	f	75	15,3	2	41	4	899,8	69,24	17,63	AD-SAA+	A+T-SAA-	p.D179H, p.E365K	E3/E3
278	f	75	15,3	3	40	4	1012	90,85	19,88	AD-SAA+	A-T-SAA+	p.E365K	E3/E3
279	m	75	16,3	2,5	17	4	728,6	66,96	20,06	AD-SAA+	A-T+SAA+	p.E365K	E3/E3
280	f	75	5,3	2	21	0				AD-SAA-	A+T-SAA-	p.E365K	E2/E3
281	m	75	7	2	31	4	687,4	81,13	23,63	AD-SAA+	A-T+SAA+	p.E365K	E3/E3
282	f	75	28,4	2	25	4	714,9	61,54	19,5	AD-SAA+	A-T+SAA+	p.L483P	E3/E3
283	f	75	29,4	4	46	4	602,3	53,29	21,19	AD-SAA+	A-T-SAA+	p.L483P	E3/E3
284	m	75	17,3	3	13	4	638,7	64,09	21,94	AD-SAA+	A-T-SAA+	p.N409S	E3/E3
285	f	75	16,3	3	30	4	1089	70,71	14,81	AD-SAA+	A+T-SAA-	p.N409S	E3/E4
286	m	75	3,1	2	11	0				AD+SAA-	A+T+SAA-	p.N409S	E3/E3
287	m	75	4,8	2	19	0				AD+SAA-	A+T+SAA-	p.N409S	E3/E3
288	m	75	11	5	68	4	491,1	59,03	24	AD-SAA+	A-T-SAA+	p.T408M	E2/E3
289	m	75	21,1	4	52	4	781,7	73,24	19,69	AD-SAA+	A-T-SAA+	p.T408M	E3/E3
290	m	75	7,3	4	23	4	852,2	77,79	20,25	AD-SAA+	A-T-SAA+	p.T408M	E2/E3
291	m	75	11,1	2	26	3	606,2	65,6	22,13	AD-SAA+	A-T-SAA+	WT	E3/E3
292	m	75	1,3	2	9	0				AD-SAA-	A-T+SAA-	WT	E3/E4
293	m	75	4,8	1	15	4	611,1	71,5	22,69	AD-SAA+	A+T-SAA-	WT	E3/E4
294	m	75	6,8	2	33	4	990,5	97,3	20,63	AD-SAA+	A+T-SAA-	WT	E3/E4
295	f	75	10	2	18	4	1092	80,5	16,5	AD-SAA+	A+T-SAA-	WT	E3/E4
296	m	75	2,1	2	20	4	745,1	65,6	22,63	AD-SAA+	A+T-SAA-	WT	E2/E3
297	m	75	9,9	3	35	4	1101	91,2	17,44	AD+SAA+	A+T+SAA+	WT	E3/E4
298	m	75	11,4	4	32	4	695,7	56,5	19,5	AD+SAA+	A+T+SAA+	WT	E3/E4
299	m	75	12,6	4	61	4	1030	66,8	15,19	AD+SAA+	A+T+SAA+	WT	E3/E4
300	m	75	13	2	47	4	792,1	62,2	18,56	AD-SAA+	A+T-SAA-	WT	E3/E3
301	m	75	7,4	2	24	4	395,5	50,9	25,69	AD-SAA+	A+T-SAA-	WT	E3/E3
302	f	75	5,6	2	22	4	805,1	77,7	19,88	AD-SAA+	A-T+SAA+	WT	E2/E4
303	f	75	4,3	1	9	4	463,6	54,9	23,81	AD+SAA+	A+T+SAA+	WT	E3/E3
304	f	75	6,1	2	27	4	544,4	59	23,06	AD+SAA+	A+T+SAA+	WT	E3/E3
305	m	75	3,5	2	31	4	673,2	70,9	21	AD-SAA+	A-T-SAA+	WT	E3/E3
306	m	75	6	2	20	4	1012	87,5	18	AD-SAA+	A-T-SAA+	WT	E3/E3
307	f	75	4,7	2	17	3	617,9	63,9	21,75	AD+SAA+	A+T+SAA+	WT	E3/E4

308	f	75	6,7	2	20	4	837,4	62,7	18,56	AD-SAA+	A-T+SAA+	WT	E3/E4
309	m	75	9,7	2,5	68	2	285,5	33	29,63	AD-SAA+	A-T-SAA+	WT	E3/E3
310	f	75	3	2	10	3	479,5	54,5	24	AD-SAA+	A-T-SAA+	WT	E3/E3
311	f	75	4	1	10	4	662,8	64,9	21	AD-SAA+	A-T-SAA+	WT	E3/E3
312	f	75	6	2	14	4	834,3	84,5	21,75	AD-SAA+	A-T-SAA+	WT	E3/E3
313	f	75	6,9	2	15	4	817,1	68,6	18,94	AD-SAA+	A-T-SAA+	WT	E3/E3
314	m	75	7,2	2	39	4	847,7	74,1	18,94	AD-SAA+	A-T-SAA+	WT	E3/E3
315	m	75	4,7	2	19	4	666,7	62	20,81	AD-SAA+	A+T-SAA-	WT	E3/E3
316	f	75	2,9	2	13	0				AD+SAA-	A+T+SAA-	WT	E3/E4
317	m	80	4,5	2	22	4	1013	83,5	17,81	AD-SAA+	A+T-SAA-	WT	E2/E3
318	f	80	8,7	2	24	4	915	68,5	17,25	AD+SAA+	A+T+SAA+	WT	E3/E4
319	m	80	7,1	2	21	4	880,2	75,6	18,56	AD-SAA+	A-T-SAA+	WT	E3/E3
320	f	80	5,1	2	7	0				AD+SAA-	A+T+SAA-	WT	E3/E4

f=female, m=male, WT=wildtype

Supplemental Figure 1: Distribution of CSF Amyloid-beta, phopho181-tau and α-Syn in $PD_{wildtype}$ and PD_{GBA1}.

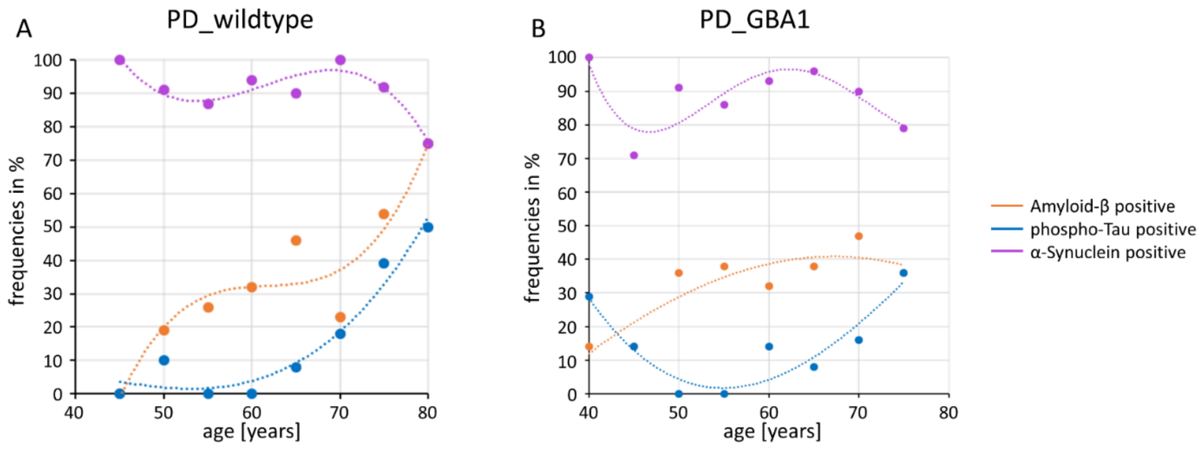

Panel A + B show the age-related distribution of CSF Amyloid-β, phospho181-Tau and α-Synuclein profiles in PDwildtype and PDGBA1. A) For $PD_{wildtype}$ presence of Amyloid-β and phopho181-Tau profiles are correlated with increased age while CSF α-Synuclein profile remained stable. B) For PD_{GBA1} the distribution of the CSF profiles did not differ between age groups.

Supplemental Figure 2: α-syn RT-QuIC fluorescence emission curves in the study cohort.

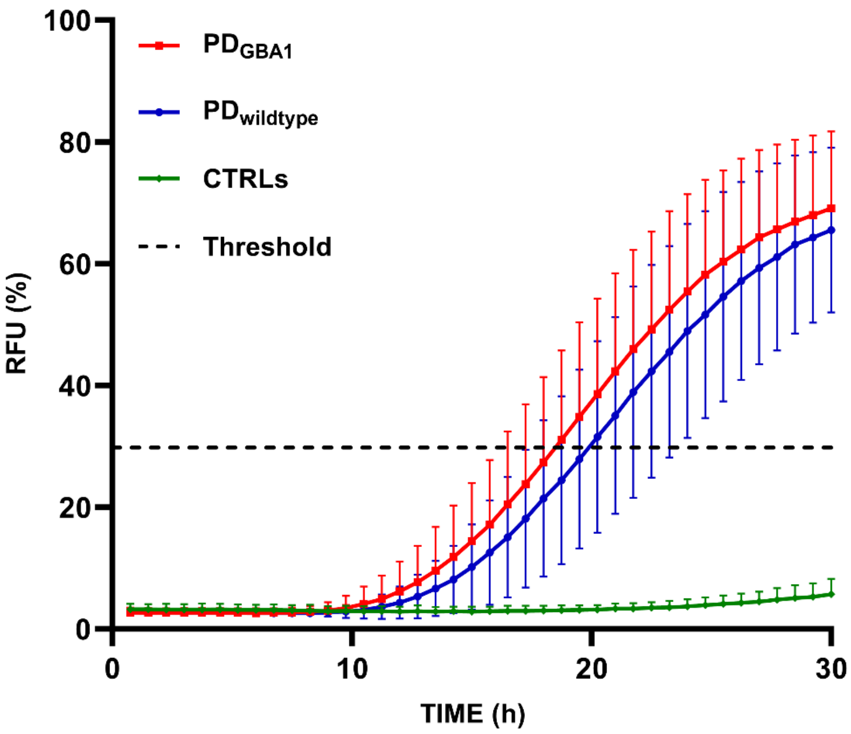

Mean normalized fluorescence emission curves of all tested cases. The PD_{GBA1}, $PD_{wildtype}$ and Controls (CTRLs) groups are shown separately. The black dashed line represents the threshold. The error bars indicate the standard deviation.
